# Clustering for Automated Exploratory Pattern Discovery in Animal Behavioral Data

**DOI:** 10.3389/fvets.2022.884437

**Published:** 2022-06-23

**Authors:** Tom Menaker, Joke Monteny, Lin Op de Beeck, Anna Zamansky

**Affiliations:** ^1^Information Systems Department, University of Haifa, Haifa, Israel; ^2^Department of Biotechnology, Vives University College, Ghent, Belgium

**Keywords:** machine learning, clustering, animal behavior, behavioral testing, Data Science

## Abstract

Traditional methods of data analysis in animal behavior research are usually based on measuring behavior by manually coding a set of chosen behavioral parameters, which is naturally prone to human bias and error, and is also a tedious labor-intensive task. Machine learning techniques are increasingly applied to support researchers in this field, mostly in a supervised manner: for tracking animals, detecting land marks or recognizing actions. Unsupervised methods are increasingly used, but are under-explored in the context of behavior studies and applied contexts such as behavioral testing of dogs. This study explores the potential of unsupervised approaches such as clustering for the automated discovery of patterns in data which have potential behavioral meaning. We aim to demonstrate that such patterns can be useful at exploratory stages of data analysis before forming specific hypotheses. To this end, we propose a concrete method for grouping video trials of behavioral testing of animal individuals into clusters using a set of potentially relevant features. Using an example of protocol for testing in a “Stranger Test”, we compare the discovered clusters against the C-BARQ owner-based questionnaire, which is commonly used for dog behavioral trait assessment, showing that our method separated well between dogs with higher C-BARQ scores for stranger fear, and those with lower scores. This demonstrates potential use of such clustering approach for exploration prior to hypothesis forming and testing in behavioral research.

## 1. Introduction

Measuring behavior is key to behavioral testing, as well as many other behavior-related research methods in ecology, neuroscience, veterinary science, psychology, and many more. Traditionally, it is done through direct observation, and involves carefully designed steps: choosing the behavioral categories to observe, defining them in precise terms (usually they can have types of either event or state), deciding on the type of measurement, sampling method, etc. The seminal book “Measuring Behavior: an Introductory Guide” by Martin and Bateson (1) provides an excellent introduction to this topic.

However, it has long been acknowledged that relying on human observation imposes severe limitations on behavioral data acquisition and analysis. As highlighted by Anderson and Perona (2), it is first of all a laborious and tedious task, limiting the volumes of processed data, as well as the number of analyzed behaviors or behavioral variables. But even more importantly, human analysis of behavior is prone to *subjectivity*. It strongly depends on human perceptual abilities, leaving lots of room for human error and making efficient tacit knowledge transfer in training. Moreover, human understanding and interpretation of behavior is in itself subjective and sometimes inconsistent.

The need for promoting objective and quantifiable assessment and measurement of behavior [cf. (3–5)] pushes forward the emerging field of computational animal behavior analysis (2, 6), in which a variety of machine learning (ML) techniques are employed for animal behavior analysis. The release of the deep learning framework DeepLabCut (7) has unleashed the potential of video-based motion tracking and pose recognition in many animal species. Additional tools such as EZtrack (8), LEAP (9), DeepPoseKit (10), idtracker.ai (11) provide more light-weight options, large group tracking and more features.

Yet even our AI-supported abilities to analyze animal behavior remain inherently human-biased in a number of aspects. First of all, most of the tools mentioned above are based on supervised learning approaches, meaning that they learn from data annotated by human experts. But humans also choose the behavioral parameters for AI to recognize, usually based on some a-priori hypotheses. As highlighted by Forkosh (12), “We can now track the position of every fly's leg or immerse a tiny fish inside a virtual world by monitoring its gaze in real time. Yet capturing animals' posture or gaze is not like understanding their behavior. Instead, behaviors are still often interpreted by human observers in an anthropomorphic manner. Even newer tools that automatically classify behaviors rely on human observers for the choice of behaviors”. Forkosh suggests focusing on animal personality as a roadmap to human-free interpretation of behavior, as personality is linked to behavior and can be quantified objectively.

Hsu and Yttri (13) refer to methods in which pre-established (by humans) criteria are applied to behavioral data as “top-down”, reiterating the problematic aspects of supervised machine learning classifiers are trained to replicate their user's annotations. They suggest *unsupervised learning algorithms* as an alternative route to overcoming this gap. Such methods allow for searching hidden patterns in data without making a-priori hypotheses or deciding on specific parameters to measure. One of the most important unsupervised learning problems is *clustering* (14, 15), which aims to find structure in a collection of unlabeled data by extracting useful features. Clustering means in a sense organizing objects into groups, the members of which share some similarity, and discovering the characteristics of this similarity. A cluster is therefore a collection of objects which are “similar” between them, and are “dissimilar” to the objects in other clusters.

A paradigm shift toward less supervised and more “human-free” automated analysis methods can recently be observed in many animal-related fields. In neuroscience, for instance a new generation of tools such as MotionMapper (16) and MoSeq (17) allow for “human-free” discovery of behaviors through clustering sophisticated motion representations and have been applied in neuroscience for the study of behaviors of mice (17), zebrafish (18), fruit flies (16), and more. A similar shift can be observed in ecology, where unsupervised approaches are applied to analyze animal movement trajectories (19–21).

While more attention is turned toward unsupervised approaches in neuroscience and ecology, this topic remains under-explored in the context of dog behavior, and specifically—behavioral testing. As a consequence of their living close to humans as pets, working or sheltered animals, dogs exhibit immense behavioral variability, stemming from their innate capacities as well as from environmental influence (22). Therefore, methods of *canine behavioral testing* are popular in research and practice. They are extensively used in cognitive science, veterinary science, working dog organizations, shelters for various purposes such as selection for breeding (23), learning abilities (24), prediction of suitability for work (25), adoptability in shelters (26), animal models for human diseases (27), welfare (28), and many more.

Machine learning approaches are only beginning to be applied in the context of canine behavioral testing. As such testing usually involves dogs freely moving in a room or outside, in naturalistic settings. Automating those approaches present additional challenge as they have mainly been applied in a “top-down” manner, i.e., for supporting manual coding and checking specific hypotheses. For instance in (29), automated analysis was used to support behavioral testing analysis in a multi-method study on canine attachment to care-giver. In (30), supervised machine learning methods were used to classify hyperactive behavior of dogs visiting a veterinary clinic.

To the best of our knowledge, the route of *unsupervised* learning in the context of behavioral testing has not yet been explored. Yet, similarly to the advantages discussed above, it has potential to reduce human bias and allow the exploration of a huge space of patterns without making a-priori hypotheses about the data. In contrast to traditional methods of data analysis in animal behavior research, where a hypothesis is made to identify parameters for coding, using unsupervised exploration one can discover many new options and combinations.

This study aims to explore this idea, providing a concrete framework for its implementation in the context of behavioral testing. Due to the exploratory nature of this research, we apply clustering techniques to *movement trajectories*, which present a simplified representation of the dog behavior during testing. These trajectories can be obtained by automated tracking, therefore providing a completely automated pipeline. We evaluate our approach on a case study of “stranger test” behavioral testing, aimed to detect aggression and fear toward strangers. We demonstrate that our approach is able to identify clusters of dogs which are aggressive and fearful toward a stranger and those who are less so, providing concrete characterization of these groups in terms of objective features related to their movement. However, these results can only be viewed as preliminary work in progress due to the small amount of samples that were available to us in this dataset, and future extension of the validation to larger datasets is needed.

The rest of this article is structured as follows. Section 2 presents our case study, which will be also used as a running example for demonstrating the different aspects of our approach: a dataset of 30 dogs, tested in a “stranger test” protocol aiming to test aggression and fear toward strangers. In Section 3, we describe the proposed clustering method and its implementation, using the “stranger test” case study as a running example. Finally, Section 4 summarizes and discusses some directions for future research.

## 2. The “Stranger Test” Case Study

Behavioral traits in animals are consistent patterns of behaviors exhibited in similar situations (31, 32). They are believed to be driven by personality (33), which is a combination of genetic, cognitive, and environmental factors (34). Assessment of personality traits in dogs is increasingly investigated due to its many practical applications in applied behavior. Some examples are determining suitability of working dogs [see, e.g., (23, 35–37)], identifying problematic behaviors (38, 39), adoption-related issues for shelter dogs (32). Jones and Gosling (40) provide a comprehensive review of past research into temperament and personality traits of dogs.

Questionnaires and rating scales are the most common way for assessing behavioral traits in dogs. The Canine Behavioral Assessment and Research Questionnaire (C-BARQ) is one of the most commonly used canine behavioral questionnaires (41, 42). However, this and other owner-administered questionnaires are very costly in terms of time both for filling and processing efforts.

The “stranger test”, developed by Joke Monteny, who also performed data collection at VIVES, Belgium, is a simple protocol aimed to test stranger-directed behavior of dogs in a simple, standardized setting. We present a short overview, while the full details of the protocol are out of scope of the current study. The test was conducted indoors, in a fenced arena, with the stranger sitting in the center in a marked, fixed location, with a GoPro video camera fixed on the ceiling, covering the whole test area.

The testing phase lasts 40 s, with the dog unleashed in the arena, and the initial contact between the dog and an unfamiliar person is recorded. No actions of the test person are performed straightly toward the dog.

Our initial dataset consists of 30 trials. The dog participants were recruited *via* social media in Belgium. The inclusion criteria were: between 1 and 2 years old, and properly vaccinated and no known health issues. The participants' owners were requested to fill a Dutch version (43) of the C-BARQ questionnaire. The questionnaire identifies the following factors, which will be used in our study: (1) Stranger directed aggression (SDA), (2) Owner directed aggression (ODA), (3) Stranger directed fear (SDF), (4) Non social fear (NSF), (5) Separation related behavior (SRB), (6) Attachment seeking behavior (ASB), (7) Excitability (EXC), and (8) Pain sensitivity (PS).

## 3. The Clustering Method

The suggested method takes as input a set of video recordings, representing behavioral testing trials of different animal individuals. Based on the testing protocol, a set of potentially relevant features are decided upon by domain experts. To make the discovery of patterns fully automatic, we assume the features can be automatically extracted from the video (we demonstrate a concrete way of doing so below). However, also manual coding could be appropriate in this context.

The method is an implementation of a commonly used data analysis pipeline based on unsupervised clustering techniques from Data Science. To build the bridge from Animal Behavior research methods to Data Science research methods, we make the observation that *behavioral parameters*, (manually or automatically) coded in behavioral studies, can be thought of as machine learning features which can be used for clustering. This shift is not only related to terminology, but is deeply rooted in different research methods in the two disciplines. While behavioral parameters are a small set, carefully chosen by human experts, usually serving to prove or refute a certain hypothesis (1), unsupervised approaches in Data Science do not assume a fixed hypothesis and do not require a-priori choice of features—there are numerous ways for automatic feature selection, some of which we employ in our approach.

**Figure 1:**
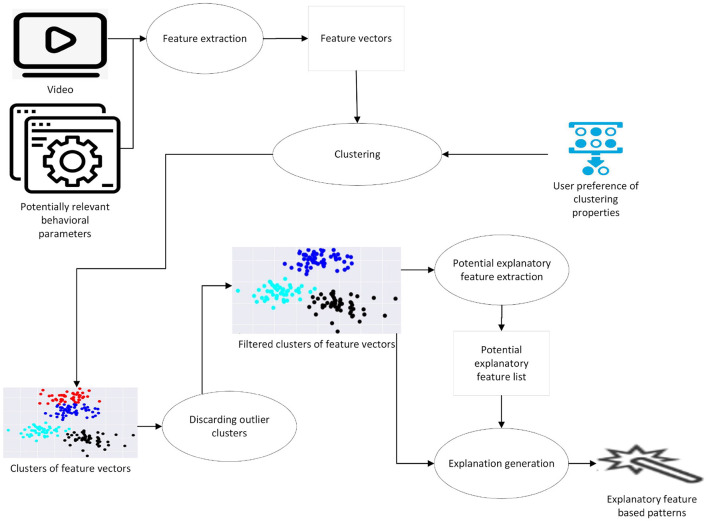
A high level overview of the general approach.

Figure 1 presents a high level overview of the pipeline, taking as input a set of video trials, and potentially relevant behavioral parameters which can be turned into features for clustering. Examples of such parameters are, e.g., trajectory length or time until a certain event in the trial. The output of the pipeline is the identification of “similarly behaving” individuals, together with a pattern: e.g., animals in cluster 1 have higher speed of movement and shorter trajectories. Such patterns can then be linked to behaviorally meaningful insights in the context of the specific protocol.

Next we describe the pipeline stages and how they are implemented in more details.

### 3.1. Feature Extraction

**Figure 2:**
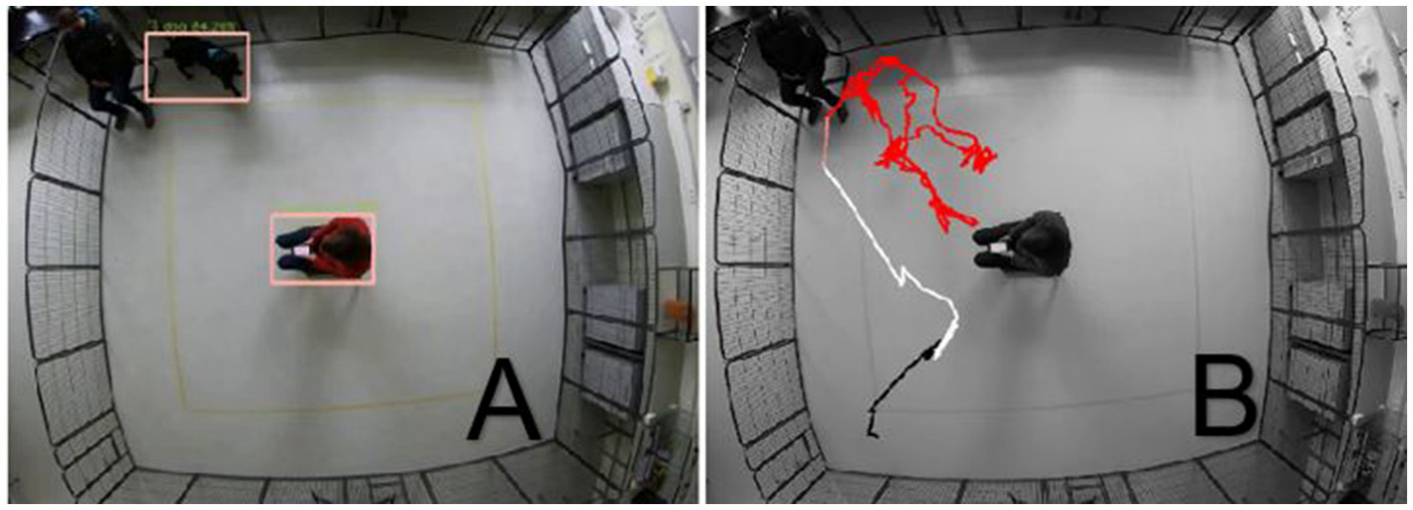
**(A)** detection; **(B)** trajectory extraction.

Building a bridge from the notion of behavioral parameters in Animal Behavior to features in Data Science, we ask: what makes a behavioral parameter a good feature? Since our main goal is to automatically produce insights into patterns found in the data, what makes a behavioral parameter a good feature is *measurability*: e.g., the availability of a method for accurately measuring the feature values for each video is important. In our case study, all of the chosen features were derived from movement trajectories, that were automatically tracked using the BLYZER tool (29, 44–46). The tool gets as input videos of trials, automatically identifies dog in a frame, and produces its movement trajectory, in the form of time series data saved in a machine-readable data (JSON format). It also has a module for computation of features from a library of available features, (such as average speed of the object, average distance between two objects, etc.). Figure 2 shows the automated detection of objects dog and stranger, and the visualized trajectory traveled by the dog in the trial.

For the purposes of our cases study, we extended this module with features identified as potentially relevant for the “Stranger Test” protocol, as shown on Table 1.

*Remarks:*

1. Start point *S* is the location of the dog at the beginning of the trial; approach point *A* is the location of the earliest point found in proximity (below a chosen threshold) to stranger; end point *F* is the location at the end of the trial.
2. Intensity of use is an animal movement metric used in (47). We decided to include this feature due to its usefulness in (30) in the context of dog behavior analysis.
3. Stranger proximity is defined as being found within a certain threshold from a circle surrounding the stranger.

**Figure 3:**
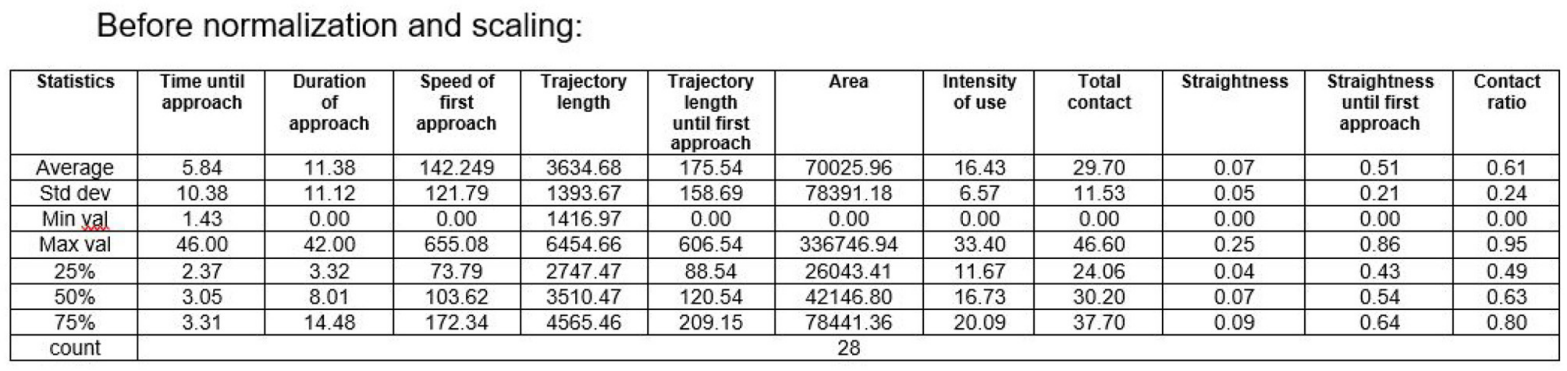
Descriptive statistics (before normalization and scaling).

Figure 3 presents some descriptive statistics of the considered features.

**Table 1:** Features for stranger test.

**Feature**	**Unit**	**Description**
Time until approach	Seconds	Time from start to first approach of stranger
Duration of approach	Seconds	Time from start of the approach to coming in close proximity to stranger
Speed of first approach	Pixels/Seconds	Average speed of first approach
Trajectory length	Pixels	Length of all traveled trajectory
Trajectory length until first approach	Pixels	Length of the trajectory from start to coming to the stranger's proximity
Area	Pixels^2^	Approximation of the area
		covered by the dog, using convex hull approximation
Intensity of use	Integer	Ratio between the total trajectory and the square root of the area covered by the trajectory
Total contact	Seconds	Time spent in proximity to stranger
Straightness	Decimal	Ratio between the distance from start point *S* to endpoint *F*, and trajectory length from *S* to *F*
Straightness until first approach	Decimal	Ratio between the distance from start point *S* to approach point *A*, and trajectory length from *S* to *A*
Contact ratio	Decimal	Percentage of frames in proximity to stranger

### 3.2. Clustering

We use one of the most commonly used clustering algorithms, k-means (48) with the usual Euclidean distance. However, applying clustering as is will result in clusters which will not be characterizable in terms of the chosen features due to the high dimensionality. To reduce the number of dimensions (see (49, 50) on common ways to deal with the curse of dimensionality), we use PCA analysis (51). PCA analysis produces linear combinations of the original variables as a set of x/y axes, also known as principal components, or PCs. Thus, after a PCA model is created, we have a set of PCs that serve as a mean to reduce the dimensionality of the original variables. In our implementation, we start first by generating all possible scenarios of dimensionality reduction using PCA. Thus, Each scenario includes a particular case of dimensionality reduction using PCA. This follows by the training of a k-means model with a discovered optimal k on the created PC's, i.e., for each PC we perform clustering with its optimal k (52). More specifically, we run each scenario described above and produce as an outcome a table with the results of the different scenarios, the table contains the following information per each scenario denoted in different rows per each scenario:

1 Amount of PC'S—the amount of PC's used for training the k-means model
2 Silhouette score—the silhouette score (53) of the trained k-means model
3 Number of clusters generated, i.e., the chosen k for the k-means model optimized by the elbow method (52)

Although we did not put any limits on the number of clusters, only scenarios where an optimal k could be found using the elbow method are included in the final list of possible clustering scenarios. Moreover, at this stage, clusters smaller than a pre-defined threshold are discarded as outliers.

#### 3.2.1. Clustering “Stranger Test” Trials

**Preprocessing**. The cut videos were pre-processed to validate the videos encoding, aspect ratio (width × height) and frames-per-second (FPS). Each video was re-encoded, using FFmpeg8^1^, with ending result of MP4 encoding, aspect ratio of 1280 × 960 and 30 FPS, respectively. Additionally, to remove noise and increase the detection rate across the video frames, post-processing operations supported by the BLYZER platform (such as smoothing and extrapolation) were applied to each video. We then used BLYZER to track the dogs movement from the videos and save their trajectory data. In the final dataset we included only trials which satisfied the following criteria: (a) The dog was identified by BLYZER with average certainty threshold above 70% across all the video frames. (b) The dog had full C-BARQ data. After this stage, 2 participants were filtered and we were left with 28 videos. Feature vectors from the pre-selected features shown on Table 1 were created for each video. They were then normalized and scaled with the standard sklearn python libraries (54).

**Clustering**. Table 2 presents the generated cluster scenario list. We only chose the first scenario for further analysis due to the maximal silhouette score, indicating a good separation between clusters, and a low number of clusters: after filtering, only two clusters were left. We present in the appendix a more in-depth analysis of the scenarios, showing plot and matrix representations of the data projections along the PCs.

**Table 2:** Generated clustering scenarios list.

**Scenario**	**PC num**	**S-score**	**Cluster** **num**	**Filtered** **cluster** **num**	**Num of samples**
**1**	1	0.537	4	2	21 - 14(c-0), 7(c-3)
**2**	2	0.324	4	3	27 - 11 (c-0), 8 (c-1), 8 (c-3)
3	3	0.272	5	2	23 - 8 (c-0), 14 (c-1)
4	4	0.255	6	3	24 - 6 (c-0), 10 (c-1), 8 (c-3)

*Bold are the chosen clusters for analysis*.

**Pattern Discovery**. In the chosen scenario, 2 clusters remained after filtering: *C*_0_ of size 14, and *C*_3_ of size 7.

The next stage is generating a list of *potential explanatory features*, i.e., includes all features that have a high (above certain threshold; we chose it as the median importance across all the features used in the PCA model) importance in the created PCs. We choose only the features that have importance above the median for at least one of the PC's in the model.

The features from the list that “explain” one or more of the clusters produce explanations (patterns) in the way formalized below. To provide intuition, for instance, in our example the explanations look as follows:

- Cluster 0 - **High** intensity of use, **High** total contact, **High** duration of approach and **High** contact ratio
- Cluster 3 - **Low** intensity of use, **Low** total contact, **Low** duration of approach and **Low** contact ratio

**Figure 4:**
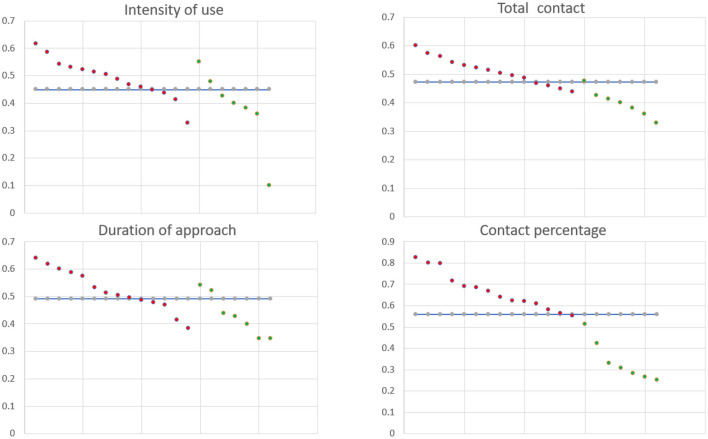
Patterns of Scenario 1: Cluster 0 red dots, Cluster 3 green dots.

**Figure 5:**
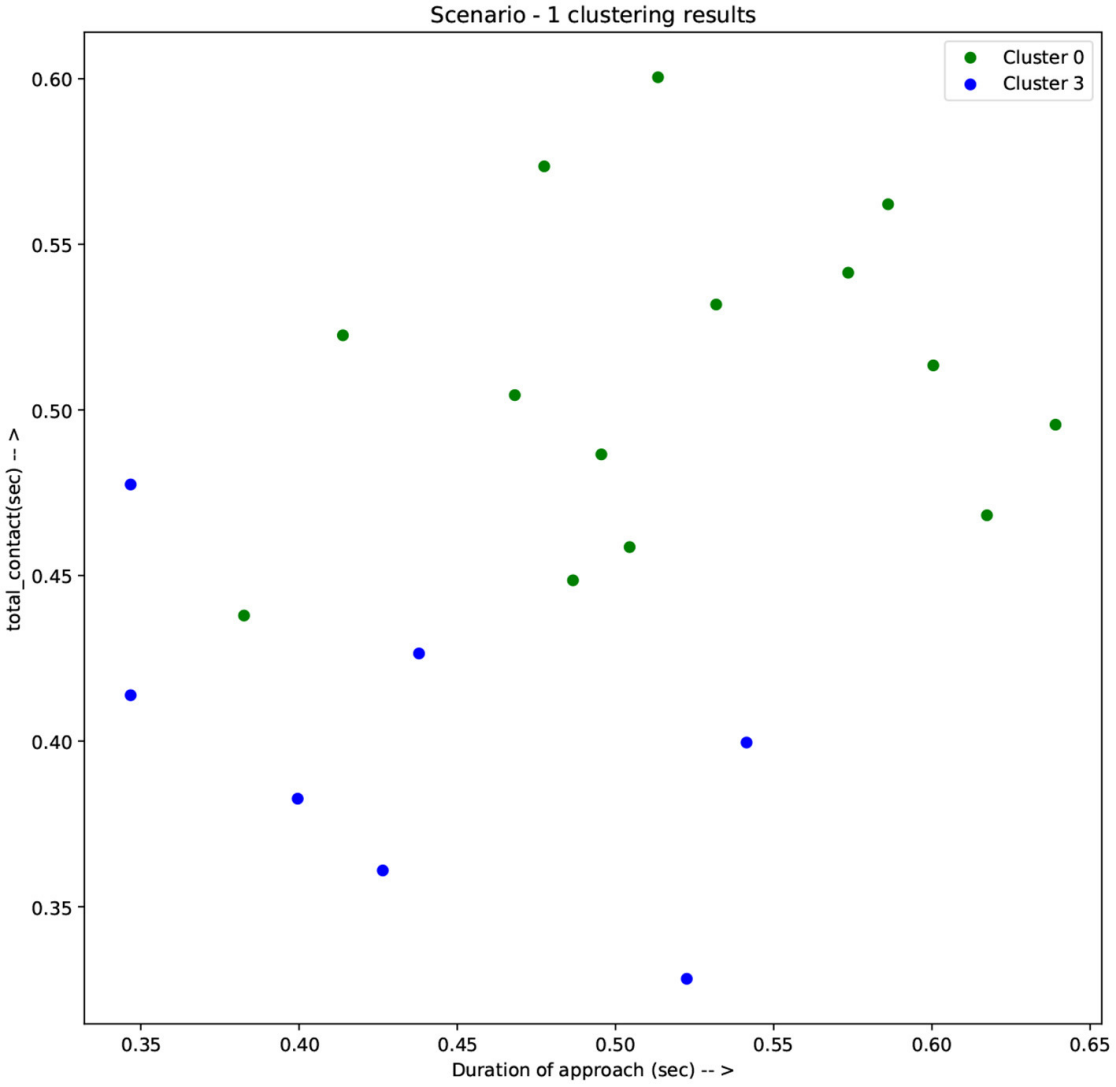
Results of clustering scenario 1 along the axes of total contact and duration of approach.

**Figure 6:**
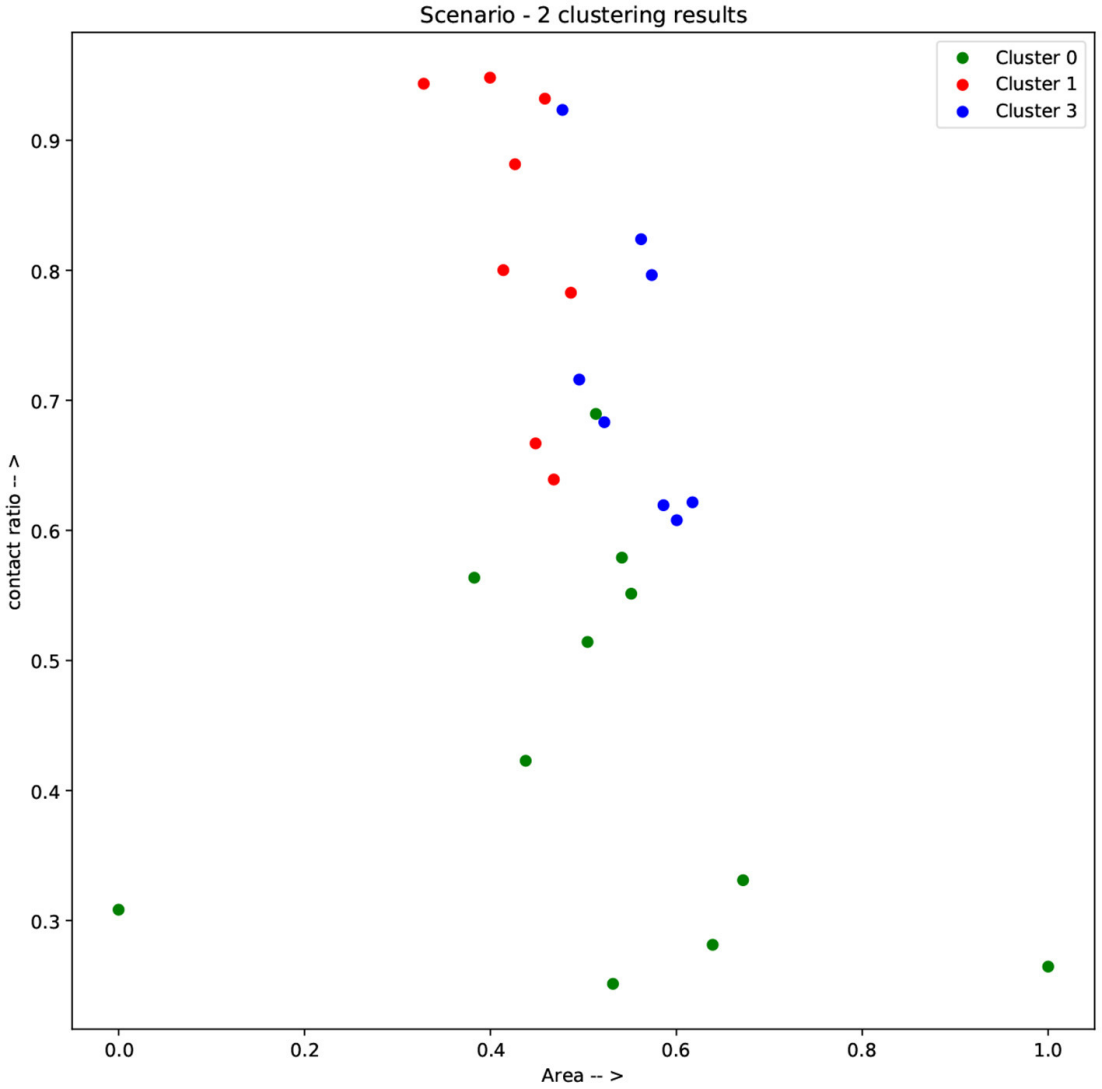
Results of clustering scenario 2 along the axes of contact ratio and area.

Figure 4 shows the distribution of the feature values among the two clusters of scenario 1, showing a good separation in terms of the four selected features. Figures 5, 6 demonstrate the clusters' distribution along two chosen explanatory features for scenarios 1 and 2, respectively.

**Formalization of explanatory features**.

The output of our approach is a list of *clustering scenarios*, i.e., suggested divisions of the samples into clusters (some samples may be discarded due to belonging to outlier clusters), together with (whenever possible) a characterization of the clusters in terms of *explanatory features*, which we define next. Intuitively, explanatory features provide an intuition for what is different in each identified cluster.

** Definition 1. (cluster)** Let *V* be a set of video samples, representing behavioral testing trials, *F* = {*f*_1_, …, *f*_*k*_} a set of features. A cluster is a subset of the set of feature vectors.

**Notation:** For a feature *f*∈*F* and a cluster *C*, we denote by *mean*(*f*)_*C*_ the mean value of *f* in *C*. For a set of clusters **C**, we denote by *mean*(*f*)_*C*_ the mean value of *f* across *C*∈**C**.

** Definition 2. (cluster separation by features and explanatory features)** Let **C** = {*C*_1_, …, *C*_*n*_} be a set of clusters over the set of feature vectors *F*(*V*). Let *C*∈**C**.

- Let *H*(*f*) be the number of samples *c*∈*C*, such that *c*_*f*_≥*mean*(*f*)_**C**_, and *L*(*f*) the number of samples *c*∈*C*, such that *c*_*f*_<*mean*(*f*)_**C**_.
- We say that *f* ↑-explains *C* if *H*(*f*)>*L*(*f*), and *f* ↓-explains *C* if *L*(*f*)>*H*(*f*). We denote by *Exp*(*f*) *H*(*f*) in the former case, and *L*(*f*) in the latter.
- We say that *f* is ↑-explanatory for *C* if *f* ↑-explains *C* and ↓-explains *C*′ for every *C*≠*C*′∈**C**.
- We say that *f* is ↓-explanatory for *C* if *f* ↓-explains *C* and ↑-explains *C*′ for every *C*≠*C*′∈**C**.
- *f* is explanatory for *C* if it is either ↑-explanatory or ↓-explanatory for it.

Intuitively, if a feature *f* is explanatory for a cluster *C*, the majority of members of C have values either higher than the rest of the clusters, or lower than the rest; thus *f* lends itself to provide a justification (or “explanation”) for *C* being chosen as a separate cluster from the rest.

**Comparison of patterns to C-BARQ**. We have considered a clustering scenario, in which well-separated clusters were found and characterized in terms of features related to objective parameters such as time until approach, trajectory length, etc. The most crucial question, however, is what behavioral meaning these clusters have, if at all. Finding an answer is highly protocol-specific, a general recipe clearly does not exist. In our case, however, we can use the C-BARQ questionnaire data for better understanding the nature of the clusters and linking them to such behavioral characteristics as fear of stranger, using the SDF and SDA factors of the C-BARQ.

**Figure 7:**
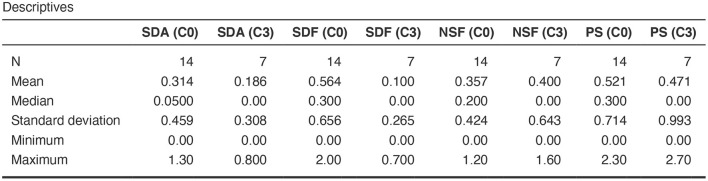
C-BARQ factors descriptive statistics for scenario 1.

**Figure 8:**
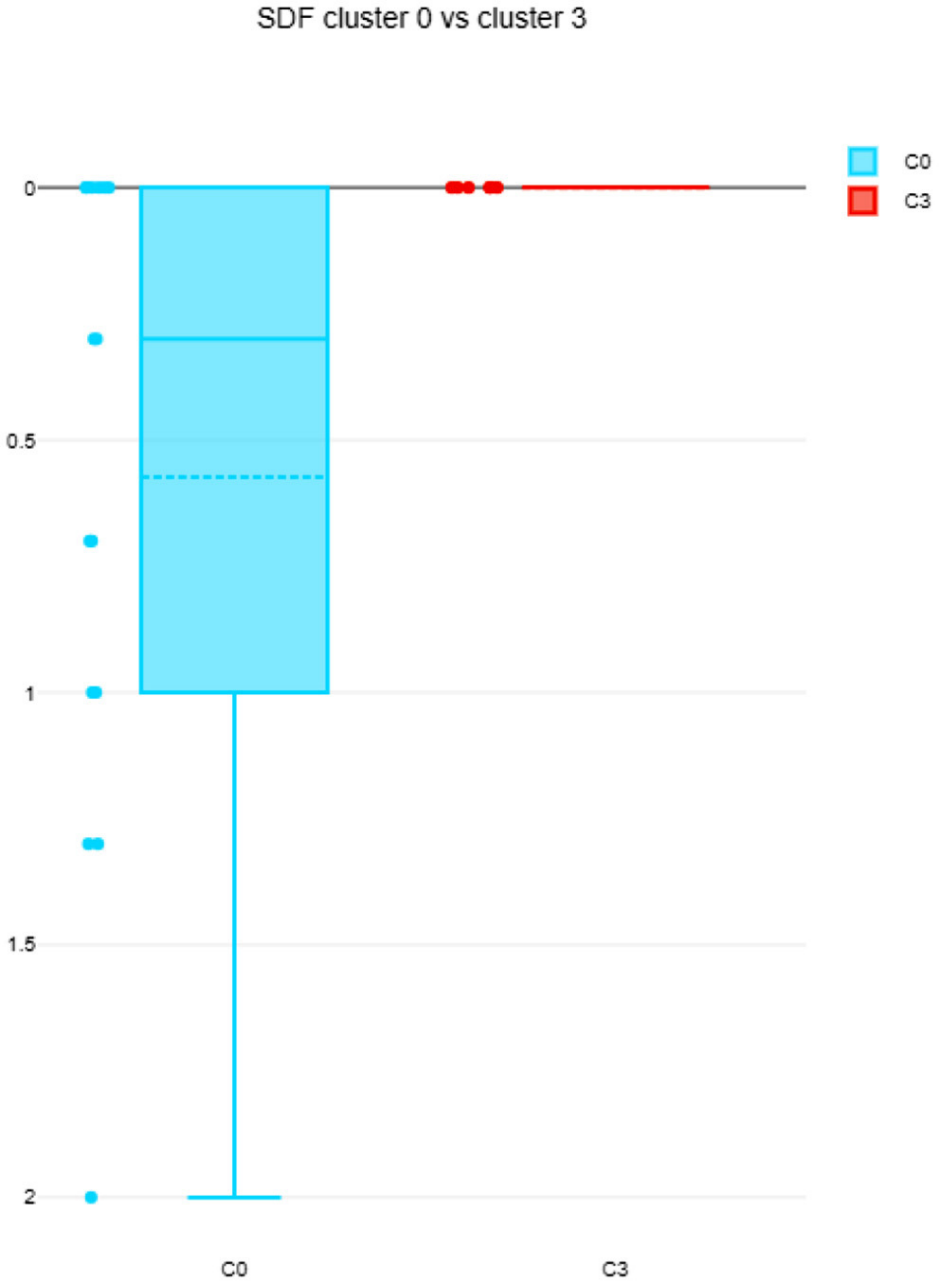
SDF comparison between C0 and C3 (scenario 1), dotted line is the median, solid line in the box is the mean.

**Figure 9:**
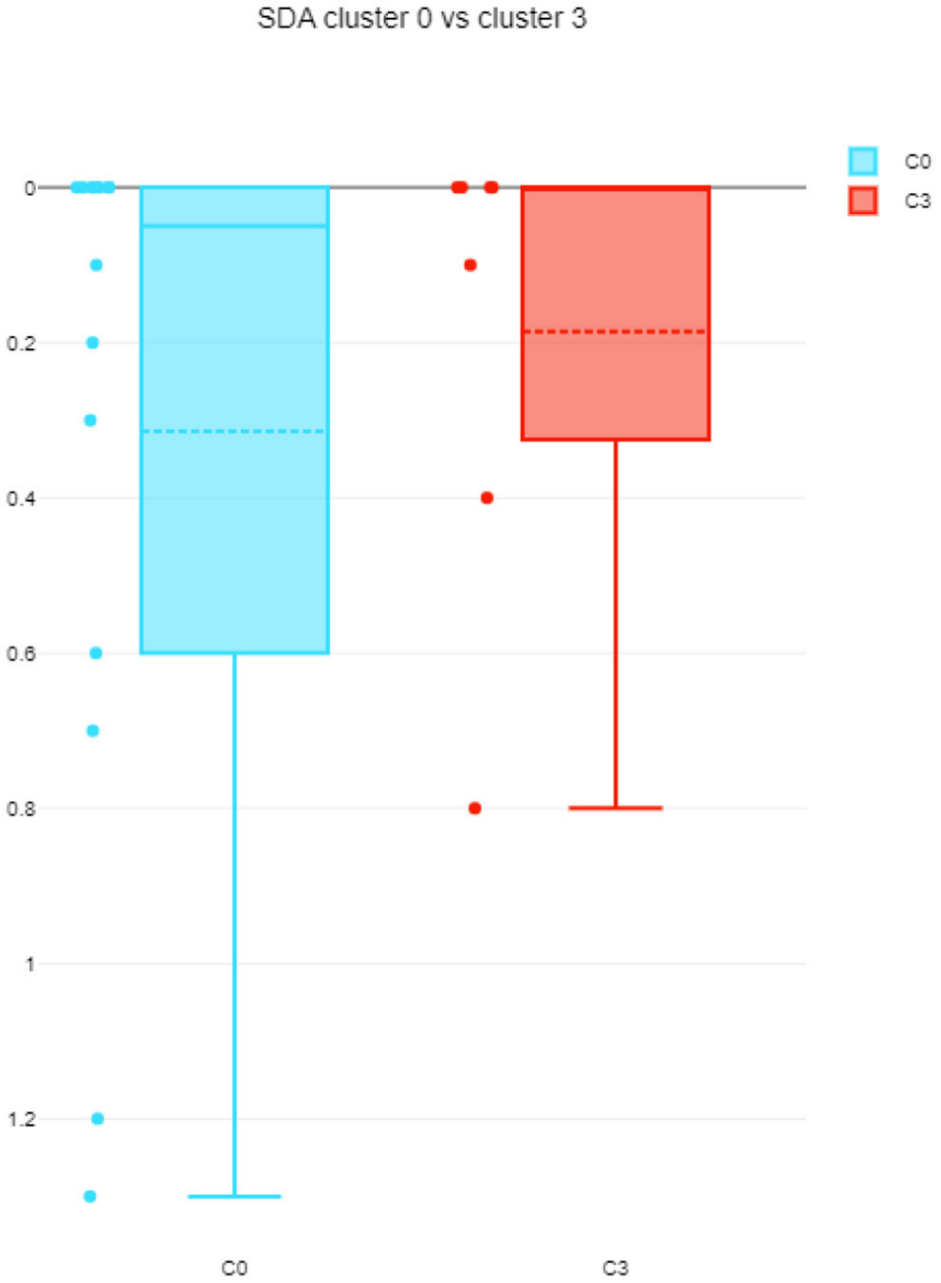
SDA comparison between C0 and C3 (scenario 1), dotted line is the median, solid line in the box is the mean.

**Figure 10:**
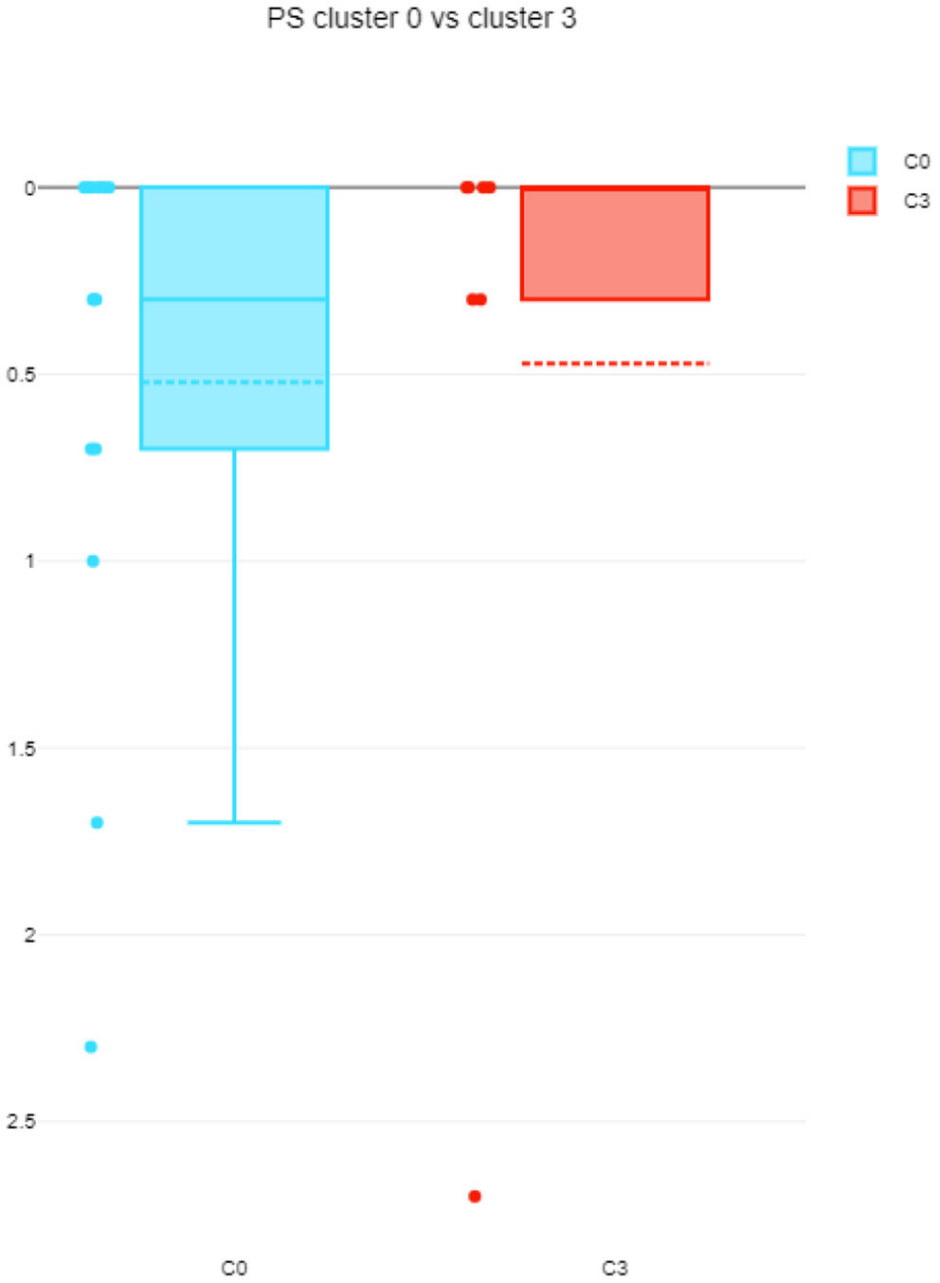
PS comparison between C0 and C3 (scenario 1), dotted line is the median, solid line in the box is the mean.

The differences among clusters were not found significant (Mann Whitney U test). This could be explained by small sample sizes. However, Figure 7 presents the descriptive statistics for the different C-BARQ factors for scenario 1 (clusters C0 and C3), from which it is evident that C0 contains dogs scoring more in SDF (stranger directed fear), SDA (stranger directed aggression), and PS (pain sensitivity) than cluster C3. While the first two factors are clearly related to stranger-related behaviors, pain sensitivity relates to fearful responses to potentially painful procedures (e.g., during veterinary examination), and is also potentially related to fearfulness. Figures 7–10 further demonstrate the differences in SDF, SDA and PS for scenario 1, the largest one being SDF. This confirms that our method separated well between dogs with higher SDF, and lower ones in terms of objective features: less dogs fearful to strangers had lower values of intensity of use, total contact, duration of approach and contact percentage. Thus, these features are potentially interesting for forming and testing further hypotheses.

## 4. Summary and Future Research

In this study we investigated the potential of unsupervised clustering techniques for discovering and explaining patterns in behavioral testing data obtained by analyzing animal trajectories. We have suggested a general approach which can be fully automatized (except for the choice of meaningful features that should be done by domain experts). Based on this framework, we implemented a method using k-means clustering algorithm, which provided a list of potentially relevant features: (1) finds “good” clustering scenarios based on commonly used metrics, and (2) generates “explanations” (characterizations) of these clusters based on these features. We evaluated the usefulness of our framework in a case study of 30 dogs tested in a “stranger test” for discovering aggression and fear toward stranger. The resulting clustering scenario discovered two clusters which were characterized by high/low intensity of use, total contact time and duration of approach. We compared the clusters against the C-BARQ owner-filled questionnaire which is a standard way for measuring stranger-directed aggression and fear, concluding that the two clusters were characterized by high/low scores on several factors of the questionnaire, specifically SDF, SDA, and PS.

Summing up, we would like to reiterate the benefits of using unsupervised clustering on trajectories of behavioral testing. Provided that a set of potentially relevant features is chosen, the method allows us to discover not only which trials are similar to which, but also in what sense they are similar, i.e., it characterizes the found similarity in terms of a small subset of the features. In the particular case of the “stranger test”, out of trajectories of 28 dogs, (in the first scenario) two clusters of 14 and 7 dogs were discovered and characterized: the former dogs contacted the stranger more and approached him quickly, while the latter dogs contacted less and approached slower. The C-BARQ data revealed that the former cluster are the dogs scoring higher with stranger directed fear and aggression. Thus the clustering method not only found a separation between these two groups, but also “explained” potential higher aggressiveness to stranger in terms of, e.g., higher speed of approach.

This provides some indication that our method was able to capture clusters that are behaviorally meaningful, and can be applied as exploratory method before forming and testing specific hypotheses concerning a behavioral testing protocol. One such exploratory finding could be that it would be important to look at speed of approach and time of contact if we are interested in aggression and fear of strangers.

We hope that this study can help promote a bridge between the disciplines of Data Science and Animal Behavior, by showing the potential use of unsupervised approaches which are under-explored in the latter discipline.

Despite the encouraging results mentioned above, it should be stressed that the low number of available samples in our dataset is a notable limitation of our study. Therefore, the clustering results cannot be viewed as validated, but rather as work in progress which requires further validation with larger number of data samples. The pipeline presented in the article, however, serves as a demonstration of the idea behind the approach, and a concrete way to implement this idea.

The results of this exploratory study open up numerous directions for future research. First of all, the k-means algorithm used in our tool can be replaced by more sophisticated methods, that will also allow for a more fine-grained analysis of the clustering outcomes. Secondly, ways to (semi)-automate the feature selection process can be explored. Thirdly, explore ways for outlier analysis and extract information from the samples that are considered as belonging to an outlier cluster, one way might be the usage of learning outlier ensembles (55, 56), Finally, we only considered here spatio-temporal data of a simple type of trajectories extracted from videos. Much more complex representations such as landmarks, segments, or fusion of audio and video data can be explored.

## Data Availability Statement

The raw data supporting the conclusions of this article will be made available by the authors, without undue reservation.

## Ethics Statement

The study was reviewed and approved by Vives University College of Roeselare, Belgium. Written informed consent was obtained from the owners for the participation of their dog in this study.

## Author Contributions

JM and LB: data collection. TM and AZ: data analysis and manuscript writing. All authors contributed to the article and approved the submitted version.

## Conflict of Interest

The authors declare that the research was conducted in the absence of any commercial or financial relationships that could be construed as a potential conflict of interest.

## Publisher's Note

All claims expressed in this article are solely those of the authors and do not necessarily represent those of their affiliated organizations, or those of the publisher, the editors and the reviewers. Any product that may be evaluated in this article, or claim that may be made by its manufacturer, is not guaranteed or endorsed by the publisher.

## Footnotes

^1^See: http://ffmpeg.org//
