# Supplementary material for: Clustering for Automated Exploratory Pattern Discovery in Animal Behavioral Data
Source: Front Vet Sci. 2022 Jun 23;9:884437. doi: 10.3389/fvets.2022.884437 (PMC9260587; doi:10.3389/fvets.2022.884437)
Supplement: Supplementary file 1 [file Data_Sheet_1.pdf]

APPENDIX: DISSECTION OF CLUSTERING RESULTS

We provide below more details from an in-depth examination of the clustering results. First, we plot the projections of the data along the eigenvectors (PCs): Figure 1 corresponds to the first scenario, presenting the projection of the data along the first eigenvector (PC1). Figure 2 corresponds to the second scenario, presenting the projection of the data along the first and the second eigenvectors (PC1,PC2). Finally, Figure 3 corresponds to the third scenario, presenting the projection of the data along the first, the second and the third eigenvectors (PC1,PC2, PC3). Secondly, we present the matrix representations of the multiplications of the data matrix with PC1 (Figure 4), PC1 and PC2 (Figure 5) and PC1, PC2 and PC3 (Figure 6).

Figure 4 using PC1 corresponds to the first scenario, which was disseminated in this paper. As can be seen, the more 'intense' features were Time until approach, Duration of approach, intensity of use, total contact, straightness until first approach and contact ratio. These constituted the list of potential explanatory features, out of which Duration of approach, intensity of use, total contact and contact ratio were chosen as explanatory features used in the analysis of scenario 1.

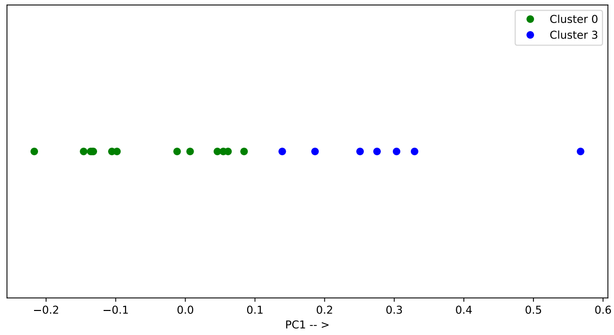

Figure 1. Projection of data on PC1 (Scenario 1)

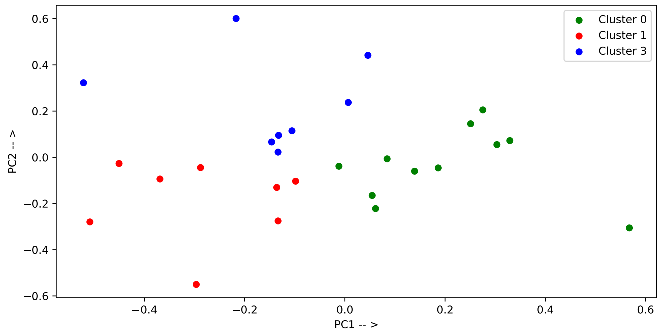

Figure 2. Projection of data on PC1 and PC2 (Scenario 2)

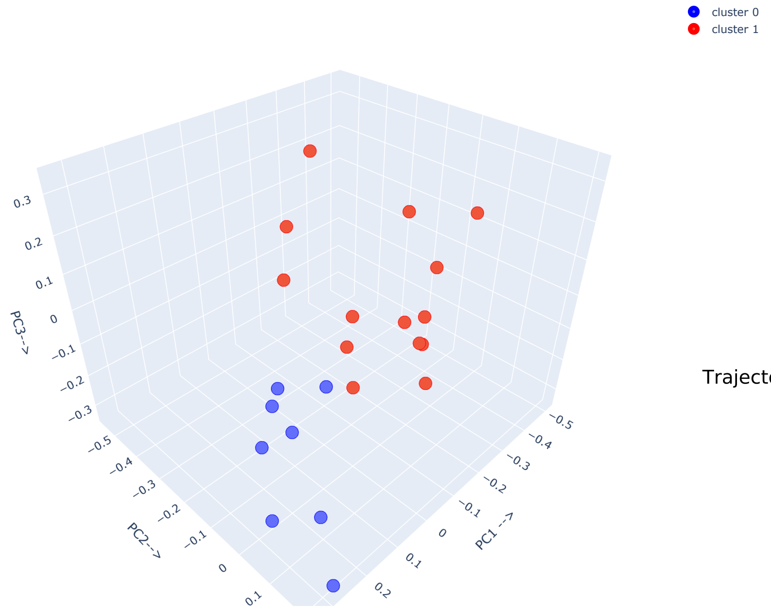

Figure 3. Projection of data on PC1,PC2 and PC3 (Scenario 3)

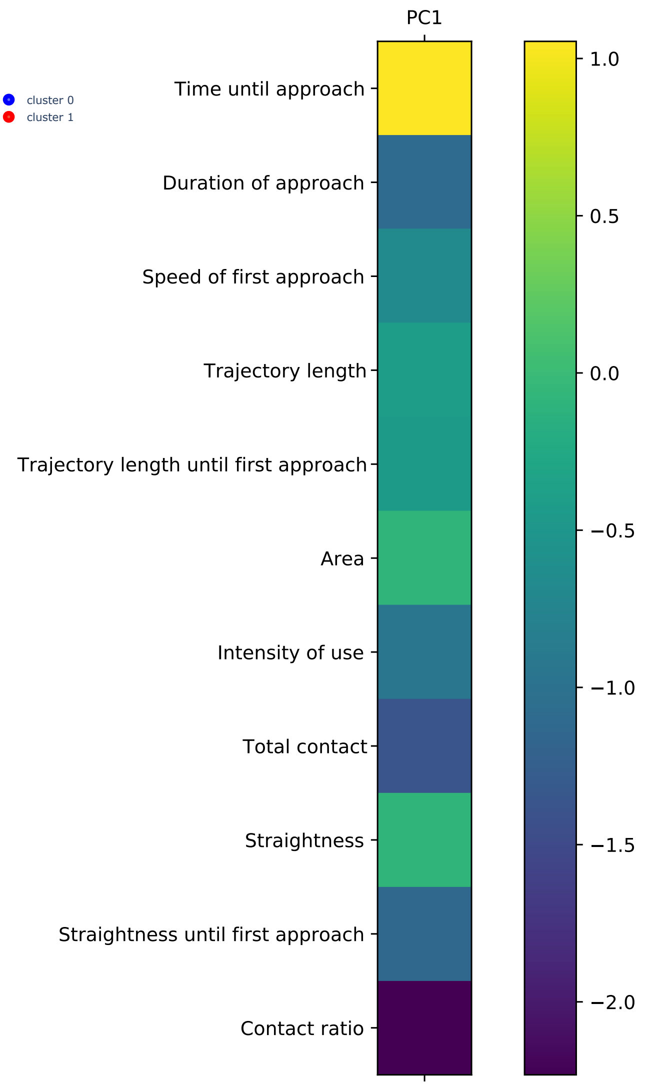

Figure 4. Matrix representation of the multiplication of the data matrix with PC1

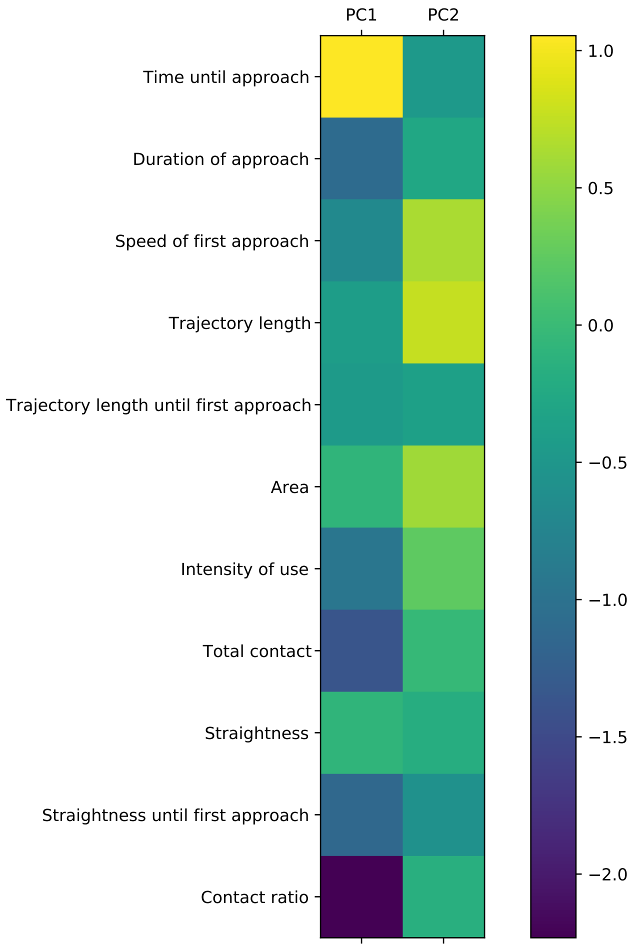

Figure 5. Matrix representation of the multiplication of the data matrix with PC1 and PC2

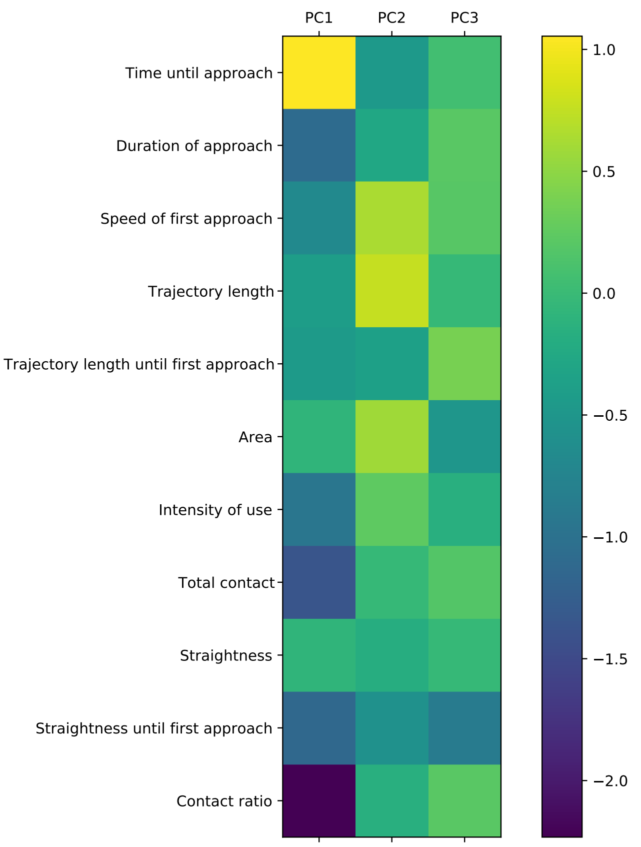

Figure 6. Matrix representation of the multiplication of the data matrix with PC1, PC2 and PC3
